# Supplementary material for: Reproducible breath metabolite changes in children with SARS-CoV-2 infection
Source: medRxiv. 2021 May 7:2020.12.04.20230755. Originally published 2020 Dec 7. Preprint. [Version 2] doi: 10.1101/2020.12.04.20230755 (PMC7743102; doi:10.1101/2020.12.04.20230755)
Supplement: Supplement 1 [file media-1.pdf]

Supplementary Material

Reproducible breath metabolite changes in children with SARS-CoV-2 infection

Amalia Z. Berna[1], Elikplim H. Akaho[1], Rebecca M. Harris[1,2,3], Morgan Congdon[1,3], Emilie Korn[1,3], Samuel Neher[1,3], Mirna M'Farrej[1,3], Julianne Burns[1,3], Audrey R. Odom John[1,3*]

[1]Department of Pediatrics, Children's Hospital of Philadelphia, Philadelphia, Pennsylvania, USA

[2]Department of Pathology and Laboratory Medicine, Children's Hospital of Philadelphia, Philadelphia, Pennsylvania, USA

[3]Perelman School of Medicine, University of Pennsylvania, Philadelphia, Pennsylvania, USA

Methods:

Thermal desorption and GCxGC parameters

Prior to analysis, sorbent tubes were brought to room temperature and loaded into autosampler (Utra-xr, Markes International, UK). A gaseous standard mixture (1.01 ppm Bromochloromethane, 1.04 ppm 1,4-Difluorobenzene, 1.04 ppm Chlorobenzene-D5, 0.96 ppm 4-bromofluorobenzene) was immediately added to each tube, followed by a purge pre-desorption step consisting of 10 min with He at 50 mL*min1, to remove water content in breath samples. Tubes were thermally desorbed for 10 min at 270°C (Unity-xr, Markes International, UK) and transferred to a "Universal" cold trap which matched the sorbent of the sample tube, held at 10°C and subsequently heated to 300°C, to minimize band broadening. The split flow after the cold trap was 15 mL*min-1.

Analysis by two-dimensional gas chromatography was conducted using an Agilent 7890B GC system, fitted with a flow modulator and a three-way splitter plate coupled to a flame ionization detector and a time-of-flight mass spectrometer with electron ionization (SepSolve, UK). Chromatographic analysis was performed using a Stabilwax (30 m × 250 µm ID × 0.25 µm df) as the first dimension (1D)-GC column and a Rtx-200 MS (5 m × 250 µm ID × 0.1 µm df) as second dimension (2D)-GC column, both purchased from Restek (Bellefonte, PA, US). The following GC oven temperature program was used: initial temperature 40°C and held for 1 min, ramped to 260°C at 3°C*min-1. The final temperature of 260°C was held for 1 min. The total run time for the analysis was 75 min. Helium carrier gas was flowed at a rate of 1.2 mL*min-1. The

flow modulator (Insight, SepSolve Analytical, UK) had a loop with dimensions 0.53 mm i.d. x 110 mm length (loop volume: 25 uL), and the modulation time was 2 s total.

TOF-MS Conditions

The GCxGC was interfaced with a BenchTOF-select time-of-flight mass spectrometer (SepSolve Analytical, UK). The acquisition speed was 50 Hz and mass range was 30-400 m/z. The ion source and transfer line were set at 250 °C and 270 °C respectively and filament voltage at 1.6 V. Electron ionization energy was 70 eV. ChromSpace (SepSolve Analytical, UK) was used to synchronize and control the INSIGHT modulator, thermal desorption, GC, and TOF.

Chemical standards and solutions

Nonanal, octanal, heptanal, tridecane, and 2-pentylfuran and isoprene were purchased from Sigma-Aldrich (St. Louis, MO, US). Dodecane was purchased from Merck (Darmstadt, Germany). To spike the compound of interest into a sorbent tube, a 10 ppm solution was prepared in HPLC grade methanol. Using a solution loading rig (Markes International Limited, UK), 1 µL of the solution was spiked into a sorbent tube. The sorbent tube was flushed for 3 min with nitrogen at a flow of 100 mL.min-1. All the stock solutions were stored in glass vials and kept at 4 °C. Sorbent tubes containing standards were analyzed by GCxGC BenchTOF-MS following the same protocols as described below for breath samples.

Quality control

Breath concentration of the canonical human volatile isoprene was performed to quality control for correct breath sampling, as a small or missing isoprene peak indicates an error in the sample collection and/or analysis, resulting in data being excluded. To check for changes in instrument sensitivity over time, a mixture of external standards was analyzed with the GCxGC BenchTOF-MS alongside the breath samples as described previously(1). Briefly, we analyzed an external standard before running each batch of breath samples. The standard used was EPA 8240B Calibration Mix (2-butanone, isobutanol, 4-methyl-2-pentanone and 2-hexanone). One mL 2000 µg.mL-1 vial standards were purchased from Sigma-Aldrich. To spike the mixture into a sorbent tube, a 100 µg.mL-1 solution was prepared in HPLC grade methanol. Using a solution loading rig (Markes International Limited, UK), 1µL of the solution was spiked into a sorbent tube. The sorbent tube was flushed for 3 min with nitrogen at a flow of 100 mL.min-1 and analyzed by GCxGC BenchTOF-MS.

Data processing and statistical analyses

Data was acquired and processed using ChromSpace (SepSolve Analytical, UK). All statistical analyses were performed using RStudio v1.3.1073 (PBC, Boston, MA) and GraphPad Prism V.8.4.3 (GraphPad Software, San Diego, CA). The workflow for data processing and statistical analysis is shown in Supplementary Figure 2. Background from the raw BenchTOF data file was removed using ChromSpace, and the Dynamic Background Compensate (DBC) of 0.2s peak width and noise factor 6.9 for typical GCxGC data was applied. DBC files were then integrated using the following parameters: peak detection deconvolution algorithm with a minimum ion count of 2000, absolute minimum peak area was set at 15,000 counts, absolute minimum peak

high was set at 10,000 counts, and no relative threshold was set for either mass height or absolute area. Compounds were given annotations using the NIST v.17 reference library. Deconvoluted peaks were exported into .xls format file. The data were then processed using RStudio to generate integrated signal for every isolated feature. Isolated features included 84 targeted volatiles, as described below.

We targeted volatiles that have been previously associated with respiratory viruses from cell culture, from analysis of *in vitro* airway cells infected with human rhinovirus, *in vivo* breath profile in swine during Influenza A infection, previously associated breath markers of COVID-19 in adults, known human body volatiles (2-8), and authors' own unpublished breath VOC library (Supplementary Table 2). The data included three internal standards (Supplementary Table 2). Chromatographic data was first normalized using internal standard (1,4-difluorobenzene), and a volatile was retained if it was present in more than 50% of the samples in either group (i.e. infected or uninfected). In total, 50 VOCs were retained and used for further statistical analysis. Unpaired Student's t-test was used to identify metabolites that were significantly different between control groups and SARS-CoV-2 groups, with a p-value of 0.05 established as the threshold for statistical significance. Of note, multiple comparison corrections of metabolomic data can increase type II errors, because metabolites are typically highly correlated and not independent features(9, 10).

Six promising breath biomarkers of SARS-CoV-2 infection were identified through volcano plot of p-value versus fold change (mean abundance SARS-CoV-2-infected/mean abundance uninfected). Principal component analysis (PCA) was used to visualize the variance between

samples in the validation set given our selection of important biomarkers. The discriminative power was assessed by ROC curve.

Supplementary Table 1. VOC biomarkers selected as best discriminants between SARS-Cov-2 positive and control (SARS-Cov-2 negative) patients, together with analytical characteristics of each compound.

Compound Name	Formula	Structure	NIST match	Rt_1	Rt_2
**Octanal**	$C_8H_{16}O$	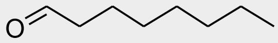	804	24.3	0.73
**Nonanal**	$C_9H_{18}O$	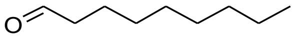	839	28.8	0.73
**Dodecane**	$C_{12}H_{24}$	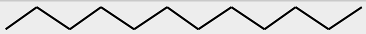	846	19.8	1.18
**Furan, 2-penthyl**	$C_9H_{14}O$	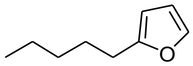	674	21.5	0.49
**Heptanal**	$C_7H_{14}O$	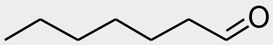	762	19.7	0.7
**Tridecane**	$C_{13}H_{28}$	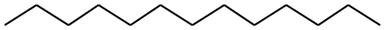	801	24.7	1.11

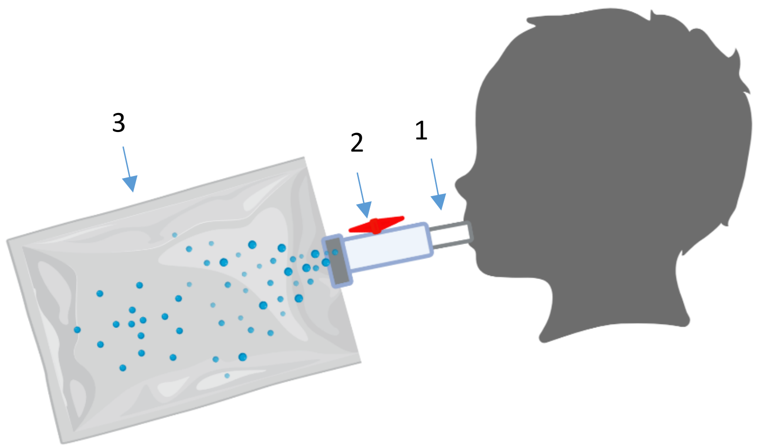

Supplementary Figure 1. Breath collection system for children. To collect breath, child places mouthpiece (1) between the lips and exhales completely. Volatiles are transferred from two-way valve (2) to SamplePro FlexFilm sample bag (3). Figure created with Biorender.com.

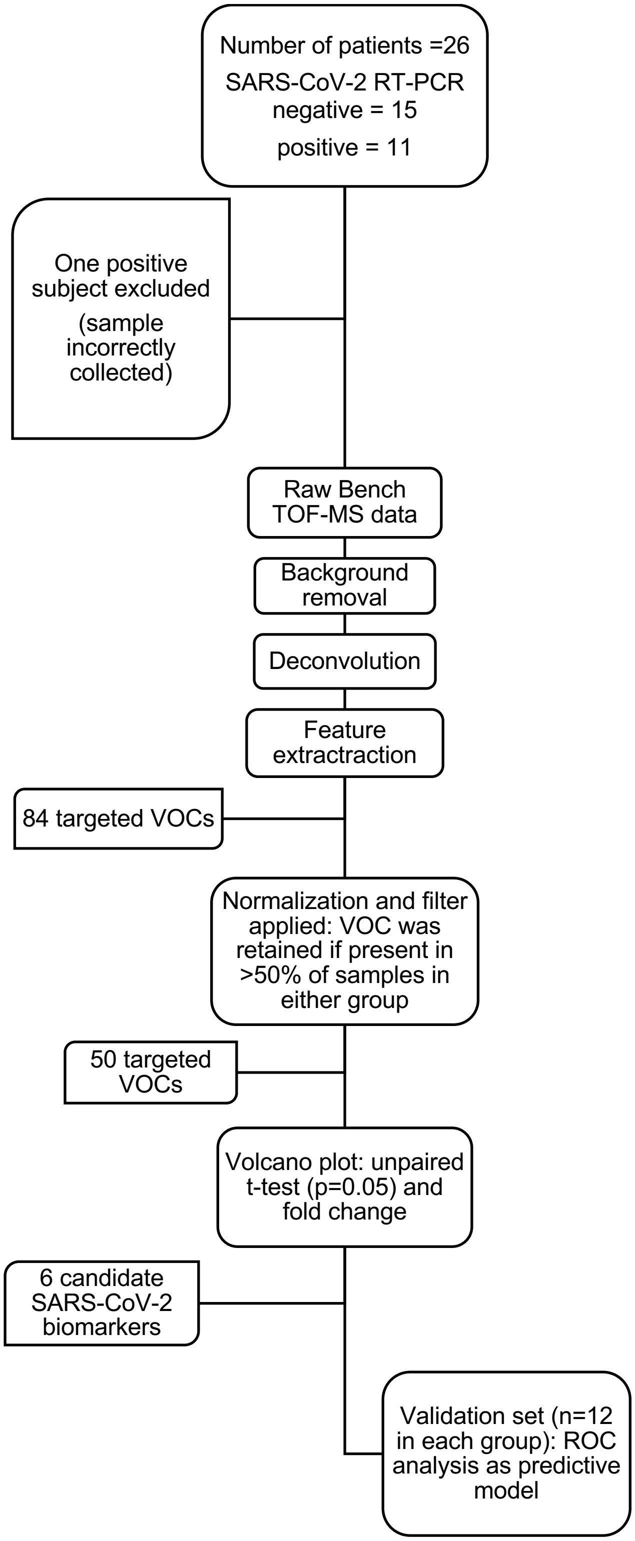

Supplementary Figure 2: Workflow of data analysis and statistics used to create a final predictive model to discriminate SARS-CoV-2-infected from -uninfected subjects.

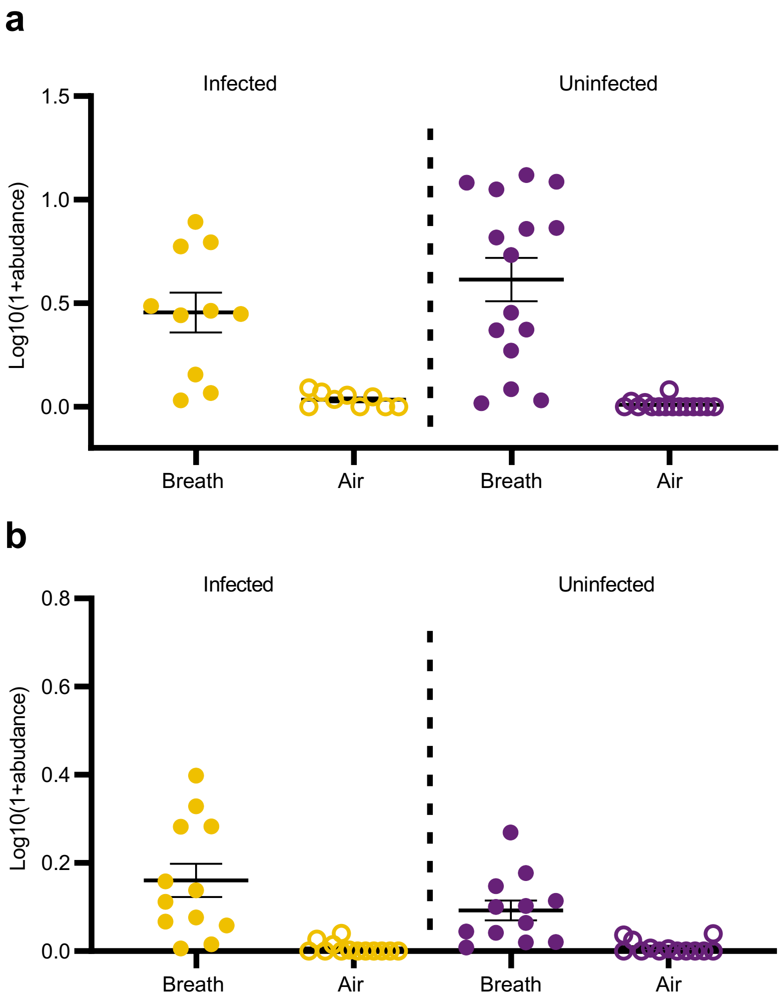

Supplementary Figure 3. Isoprene is significantly more abundant in breath samples compared to room air [discovery cohort (a) and validation cohort (b)]. Higher levels of isoprene were found in breath compared room air in both SARS-CoV-2-infected and -uninfected breath samples. Median with SEM are shown. Note that the sensitivity of the instrument changed between the time of analysis of each cohort, as a result of system maintenance and recalibration.

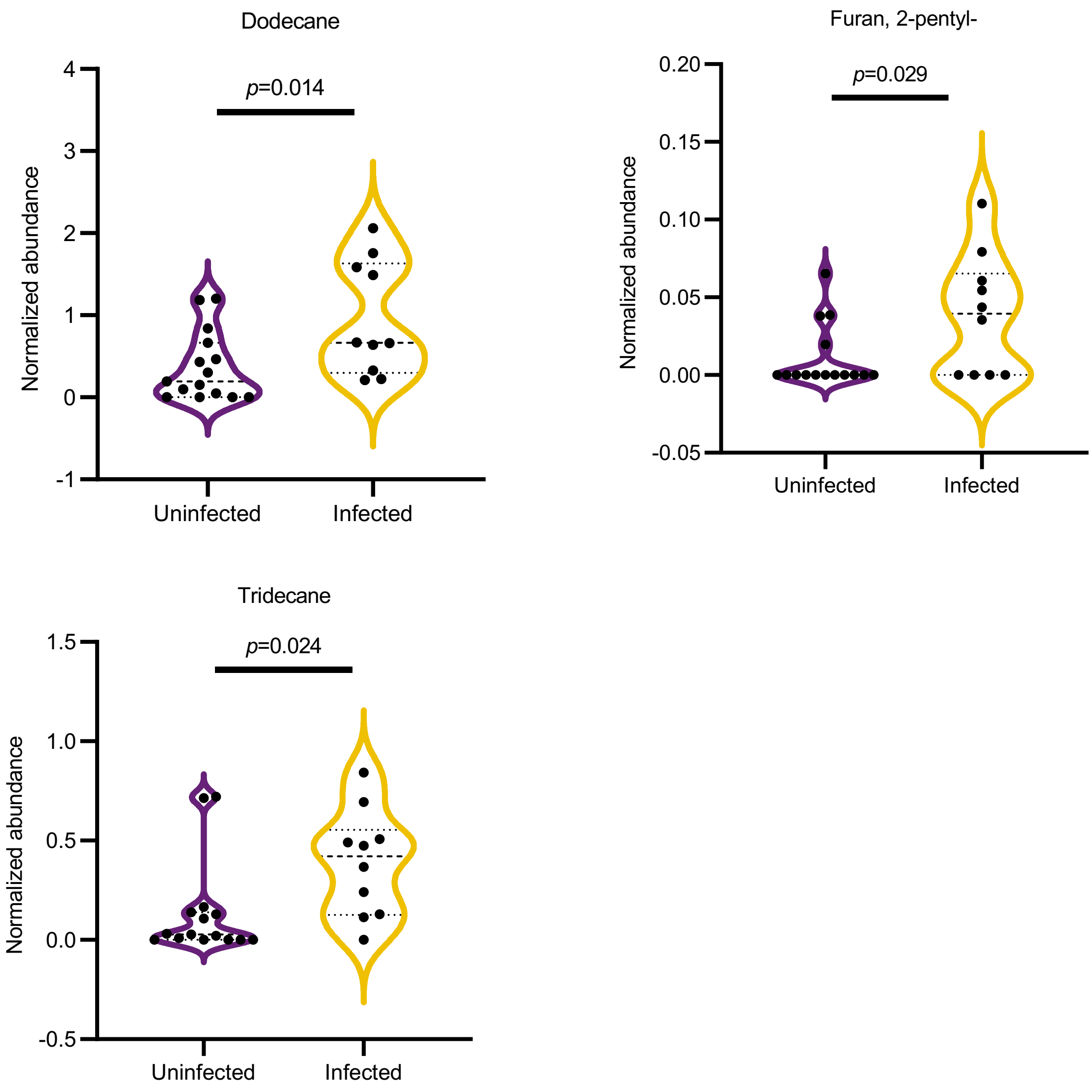

Supplementary Figure 4. Breath abundance of candidate SARS-CoV-2 biomarkers in the breath of uninfected and infected children (discovery cohort). Median and quartiles are shown. P-values from t-tests are shown for each comparison.

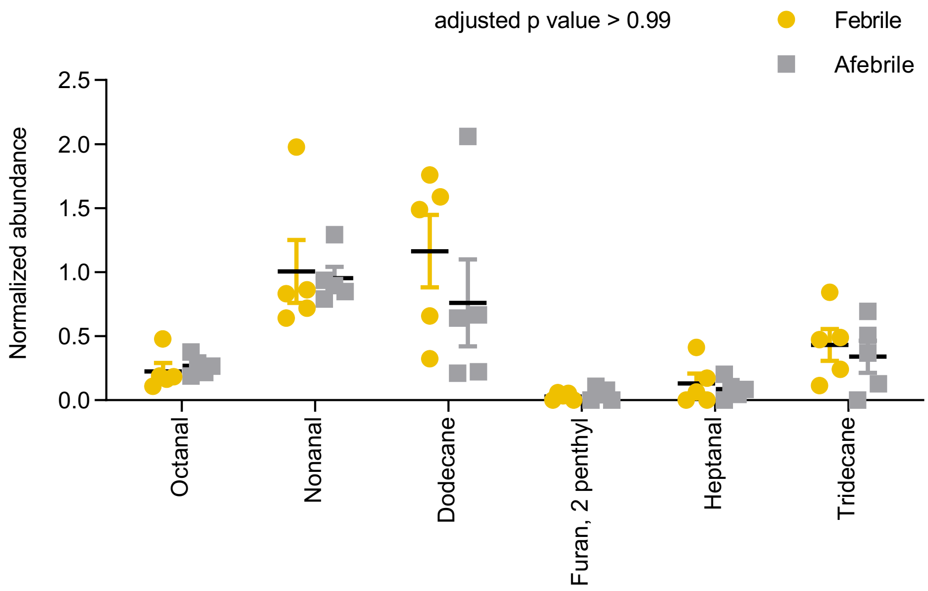

Supplementary Figure 5. Breath abundance of candidate SARS-CoV-2-associated biomarkers are not significantly different between febrile and afebrile SARS-CoV-2 patients (discovery cohort). Median with SEM are shown. Adjusted p value (t-test) for all comparisons >0.99.

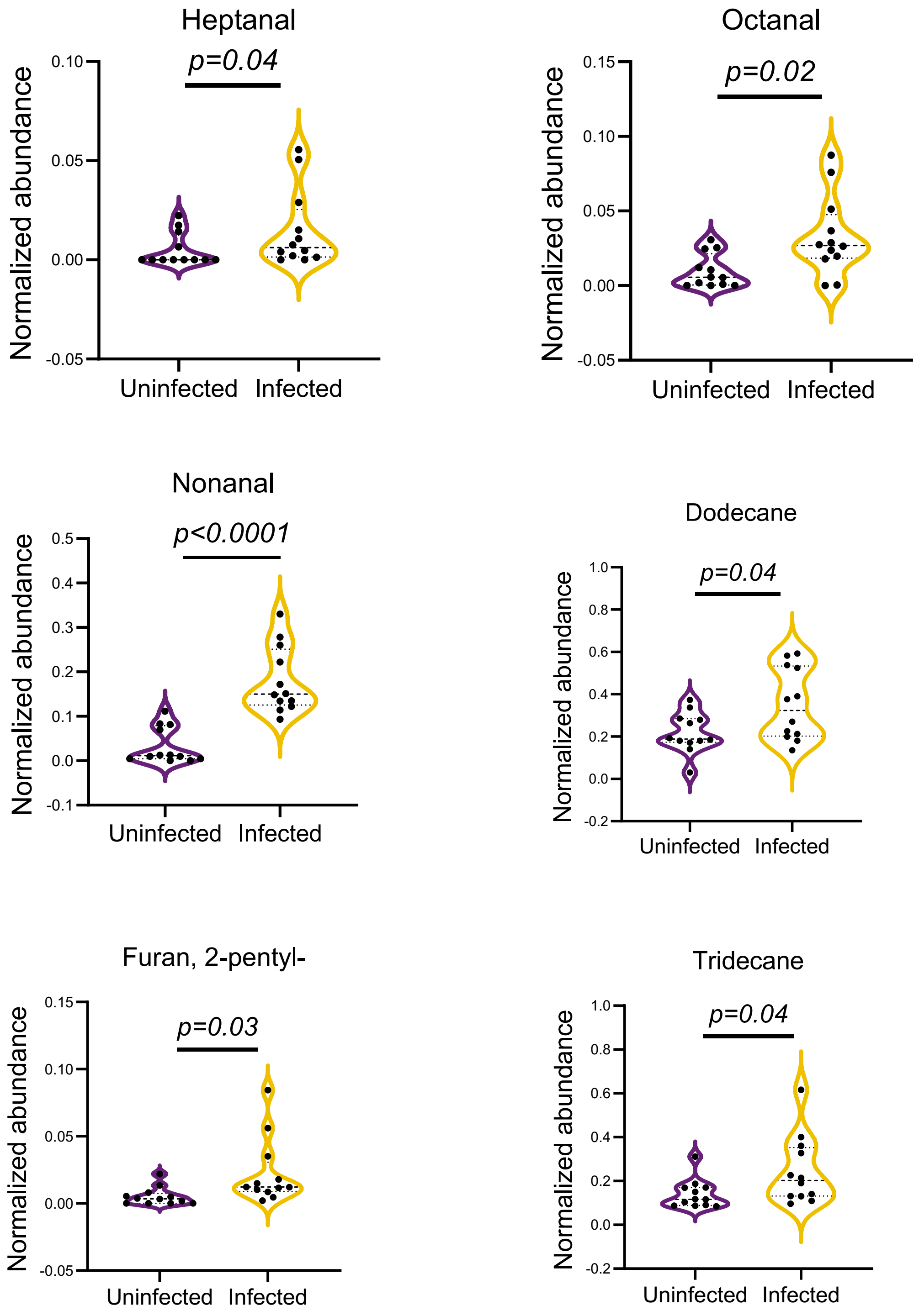

Supplementary Figure 6. Breath abundance of candidate SARS-CoV-2 biomarkers in uninfected and infected children in the validation cohort. Median and quartiles are shown. P-values (t-tests) are shown for each comparison.

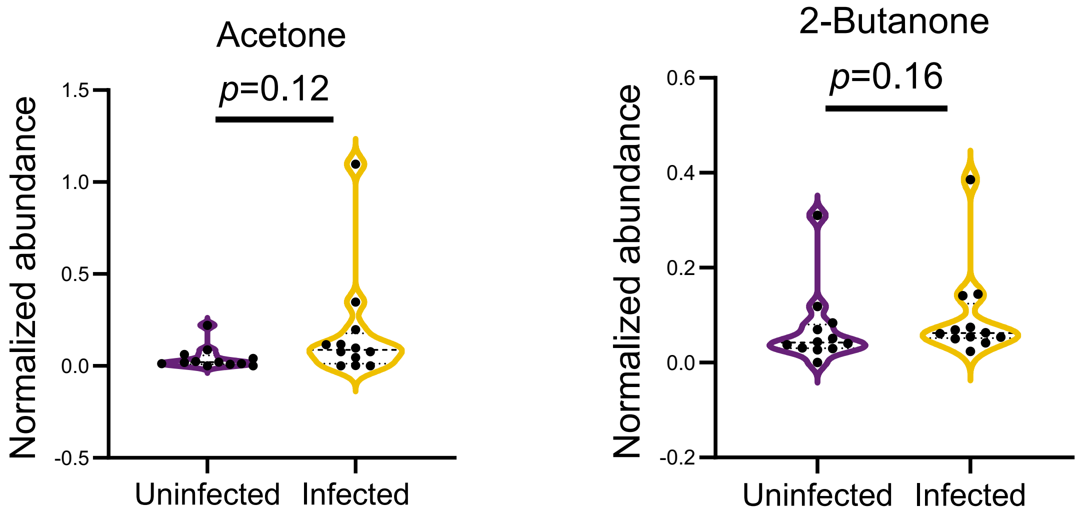

Supplementary Figure 7. Ketones do not characterize the breath of pediatric SARS-CoV-2. Acetone and 2-butanone previously reported in the breath of adults with and without COVID-19 10 were detected in our pediatric samples; however no significant differences were found between SARS-CoV-2 infected and uninfected pediatric samples in the validation cohort (similar to results in discovery cohort, Fig. 5). Median and quartiles are shown. P-values from t-tests are shown for each comparison.

References

1. Wang XR, Cassells J, and Berna AZ. Stability control for breath analysis using GC-MS. *J Chromatogr B.* 2018;1097-1098:27-34.
2. de Lacy Costello B, Amann A, Al-Kateb H, Flynn C, Filipiak W, Khalid T, et al. A review of the volatiles from the healthy human body. *J Breath Res.* 2014;8(1):014001.
3. Aksenov AA, Sandrock CE, Zhao W, Sankaran S, Schivo M, Harper R, et al. Cellular scent of influenza virus infection. *Chembiochem.* 2014;15(7):1040-8.
4. Purcaro G, Rees CA, Wieland-Alter WF, Schneider MJ, Wang X, Stefanuto PH, et al. Volatile fingerprinting of human respiratory viruses from cell culture. *J Breath Res.* 2018;12(2):026015.
5. Schivo M, Aksenov AA, Linderholm AL, McCartney MM, Simmons J, Harper RW, et al. Volatile emanations from in vitro airway cells infected with human rhinovirus. *J Breath Res.* 2014;8(3):037110.
6. Traxler S, Bischoff AC, Sass R, Trefz P, Gierschner P, Brock B, et al. VOC breath profile in spontaneously breathing awake swine during Influenza A infection. *Sci Rep.* 2018;8(1):14857.
7. Traxler S, Barkowsky G, Sass R, Klemenz AC, Patenge N, Kreikemeyer B, et al. Volatile scents of influenza A and S. pyogenes (co-)infected cells. *Sci Rep-Uk.* 2019;9.
8. Ruszkiewicz DM, Sanders D, O'Brien R, Hempel F, Reed MJ, Riepe AC, et al. Diagnosis of COVID-19 by analysis of breath with gas chromatography-ion mobility spectrometry - a feasibility study. *EClinicalMedicine.* 2020;29(100609).
9. McDonald JH, and Delaware Uo. *Handbook of Biological Statistics.* Sparky House Publishing; 2009.

10. Xi B, Gu H, Baniasadi H, and Raftery D. Statistical analysis and modeling of mass spectrometry-based metabolomics data. *Methods Mol Biol.* 2014;1198:333-53.
